# Sensory coding accuracy and perceptual performance are improved during the desynchronized cortical state

**DOI:** 10.1038/s41467-017-01030-4

**Published:** 2017-11-03

**Authors:** Charles B. Beaman, Sarah L. Eagleman, Valentin Dragoi

**Affiliations:** 1 Department of Neurobiology and Anatomy, McGovern Medical School, University of Texas at Houston, Houston, TX 77030 USA; 2 Department of Electrical and Computer Engineering, Rice University, George R. Brown School of Engineering, Houston, TX 77005 USA

## Abstract

Cortical activity changes continuously during the course of the day. At a global scale, population activity varies between the ‘synchronized’ state during sleep and ‘desynchronized’ state during waking. However, whether local fluctuations in population synchrony during wakefulness modulate the accuracy of sensory encoding and behavioral performance is poorly understood. Here, we show that populations of cells in monkey visual cortex exhibit rapid fluctuations in synchrony ranging from desynchronized responses, indicative of high alertness, to highly synchronized responses. These fluctuations are local and control the trial variability in population coding accuracy and behavioral performance in a discrimination task. When local population activity is desynchronized, the correlated variability between neurons is reduced, and network and behavioral performance are enhanced. These findings demonstrate that the structure of variability in local cortical populations is not noise but rather controls how sensory information is optimally integrated with ongoing processes to guide network coding and behavior.

## Abstract (web summary)

Changes in synchrony of cortical populations are observed across the sleep-wake cycle, however the effect of fluctuations in synchrony during wakefulness is not understood. Here the authors show that visual cortical neurons have improved sensory encoding accuracy as well as improved perceptual performance during periods of local population desynchrony.

## Introduction

The dynamics and responsiveness of populations of brain cells in alert animals vary widely across different behavioral contexts^1–5^. Thus, even in the absence of external stimulation, the state of the brain can fluctuate between synchronized activity in quiescent animals and highly desynchronized activity during alertness^6–8^. Although the large changes in brain activity and transitions between sleep and waking have been well characterized^9–11^, the functional impact of local fluctuations in population activity during alertness has remained elusive. Indeed, even though global fluctuations in brain state induced by factors such as arousal or attention have been documented^12, 13^, whether and how rapid changes in local population activity during alertness influence both the capacity of networks of neurons to encode sensory information and the behavior of the animal remains mysterious. How do fluctuations in the synchrony of local population spiking activity impact the variability in sensory coding and perceptual performance?

We examined the functional impact of trial-by-trial fluctuations in population synchrony by recording spiking activity from multiple neurons in the visual cortex (area V4), while monkeys engaged in an image orientation discrimination task. We found that the firing rates and pairwise correlated variability were impacted by the state of neuronal populations before stimulus presentation. These changes directly influenced the amount of information encoded in population activity and the animal’s behavioral performance. Specifically, neuronal and perceptual discrimination performance were enhanced when the population of cells was in a desynchronized state and were impaired when the population was in a synchronized state. The fluctuations in population synchrony that we captured were local as they were uncorrelated with global fluctuations in brain state measured by electroencephalogram (EEG) electrodes and pupil size. These results demonstrate that intrinsic fluctuations in the degree of synchrony of local visual cortical networks during wakefulness significantly influence the amount of information encoded by neuronal populations and perceptual performance.

## Results

### Trial fluctuations in population synchrony

**Fig. 1:**
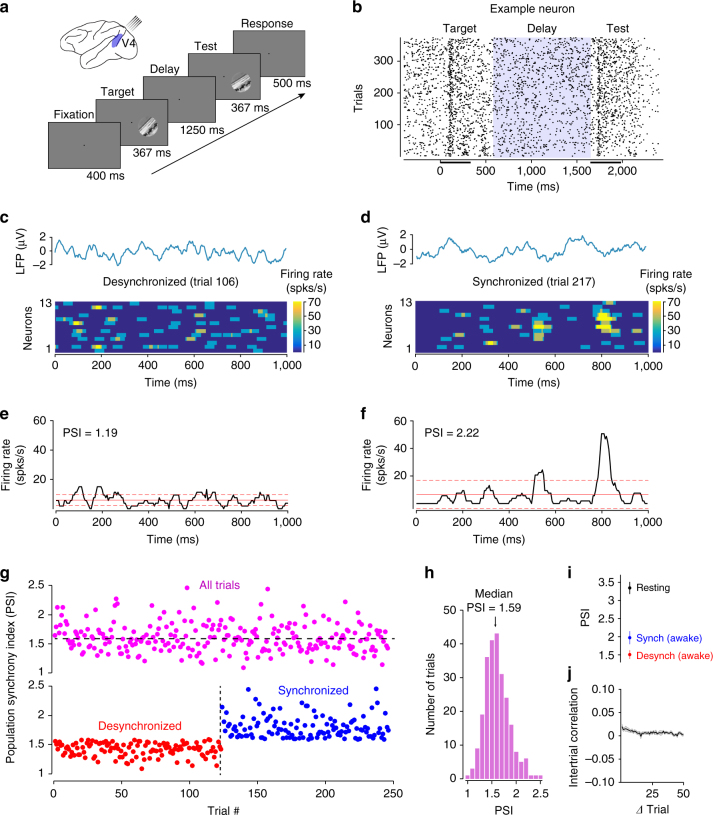
Trial-by-trial fluctuations in population synchrony in visual cortex. **a** Schematic representation of the recording site and experimental design. Animals were trained to report whether two briefly flashed successive natural scenes (target and test) were identical or different. **b** Raster plot of one example neuron. The blue shaded inter-stimulus delay period was used to measure population synchrony in each trial. The black bars under the *x* axis mark the time intervals when the two stimuli are presented. **c**, **d** Population response measured in individual trials from the same session—the neural population is desynchronized in trial 106 **c** and synchronized in trial 217 **d**. The blue traces represent local field potential responses from the individual trials. **e**, **f** Population firing rate as a function of time for the example trials in **c** and **d**. The population of cells is desynchronized in trial 106 (PSI = 1.19, **e**) and synchronized in trial 217 (PSI = 2.22, **f**). The solid red line indicates the population mean firing rate; the red dotted lines indicate 1 s.d. from this mean. **g** top Trial-by-trial PSI for the example session from **c**–**f**. The horizontal line represents the median PSI value. Bottom: Trial-by-trial PSI after dividing the session into desynchronized (PSI below median) and synchronized trials (PSI above median). **h** PSI histogram for the example session in **c**–**g**. **i** Average PSI across sessions for desynchronized (red) and synchronized trials (blue). These PSI values are compared to the mean PSI when animals rest for a 20–30 min period (black). Error bars represent standard error. **j** The mean autocorrelation function (across sessions) of trial-by-trial PSI

Two monkeys performed an image orientation discrimination task (*n* = 28 sessions), while multiple neurons (up to 17) were recorded simultaneously from visual cortical area V4. We analyzed the activity of 176 visually responsive neurons. In each trial, two identical, circular natural scenes (target and test, 8–10° in diameter) were flashed for 367 ms each, and were separated by a 1,250 ms delay (Fig. 1a). The test image was rotated by 0° (match condition), or 2°, 3°, 5°, 10° or 20° (non-match condition) with respect to the target. Both stimuli fully covered the receptive fields of the neurons recorded simultaneously in each session. The animals were trained to signal whether the two successive images were identical or different (see Methods).

We observed that even during actively engaged behavior, many trials were associated with strong fluctuations in cortical population activity ranging from desynchronized to synchronized state (Fig. 1c–f and Supplementary Fig. 1). In the example trials in Figs. 1c, d, the responses of 13 neurons are represented during the delay period between the target and test stimuli. Individual trials reveal desynchronized population responses (e.g., trial 106), while other trials (e.g., trial 217) reveal synchronized responses (i.e., distinct periods of high and low activity, Fig. 1e, f). We measured the duration of synchronized ‘high’ states (bursts) by examining the times for which the population firing rate increase was above 1 s.d. of the mean rate. Overall, we found a diversity of burst durations and frequencies across trials—synchronized bursts lasted between 20 and 100 ms, and there were 0–10 bursts per second in each trial (excluding the transient bursts lasting less than 20 ms).

To quantify the degree of synchrony in each trial, we calculated the population synchrony index (PSI, see Methods)^14, 15^, measured during the delay period between the target and test stimuli (Fig. 1b). PSI is defined as the coefficient of variation (standard deviation divided by mean) of the average population firing rate (Fig. 1) in a given time interval using 10 ms bins (we reasoned that small bins can better capture rapid fluctuations in the population response). PSI has clear advantages over other methods of synchrony, such as pairwise correlations, because it relies on the entire population, not just two cells, and can be computed in each trial. If the cells’ responses are synchronous, the standard deviation of the binned mean population response will increase (because the binned firing rate of the population will fluctuate between low and high response states, Fig. 1f), and PSI will increase. If, on the other hand, cells are desynchronized, the standard deviation of the binned population response will be lower (the difference between the low and high states of the mean population response will decrease in amplitude, Fig. 1e), which causes PSI to decrease.

Individual trials recorded in the same session differed widely in their degree of synchrony of the population response (Fig. 1g, top depicts the trial-by-trial PSI throughout this example session). We separated the trials into two groups, desynchronized and synchronized, based on whether PSI was lower or higher than the median PSI in each session (Fig. 1g, bottom). Even though we found a continuum of population synchrony states across trials—PSI values in each session were normally distributed rather than reflecting a bimodal distribution (Fig. 1h represents the PSI trial distribution for the session corresponding to Fig. 1g). To calibrate our population synchrony values with those measured in other behavioral states, we verified that the mean PSI during wakefulness is significantly lower, both in the desynchronized and synchronized states, than the resting state when animals were sitting quietly in the dark with their eyes closed (*n* = 6 sessions; desynchronized: *P* < 0.0005; synchronized: *P* < 0.01, Wilcoxon rank-sum, Fig. 1i). Across our 28 sessions, we failed to find a temporal structure in the trial fluctuations of population synchrony. The autocorrelation of the trial-by-trial PSI values revealed that across sessions the state of synchrony during wakefulness fluctuates randomly (*r* < 0.05 for all trial intervals, Fig. 1j and Supplementary Fig. 2).

One could argue that the 1 s delay period used to define cortical state does not constitute a true ‘baseline’ or ongoing response in the context of our behavioral task since the animal has to retain in its working memory the orientation of the first stimulus during the delay. However, we failed to find evidence for elevated firing (a neural correlate of working memory) during the delay period compared to the 400 ms fixation interval before the target presentation. (*P* > 0.1, Wilcoxon sign ranked test, *n* = 176 cells). Nonetheless, we directly compared population synchrony during the delay and the 400 ms fixation period. Across sessions, we found a mean correlation coefficient of *r* = 0.11 ± 0.02—i.e., out of 28 sessions, less than half (*n* = 12) were associated with a significant correlation (*P* < 0.05, Pearson correlation). However, when we extended the pre-target ongoing activity to 2 s (this time period included 1.6 s of spontaneous activity with uncontrolled eye movements), the correlation coefficient increased to *r* = 0.20 ± 0.04—18 out of 28 sessions showed a significant correlation.

### Cortical state influences encoded information

**Fig. 2:**
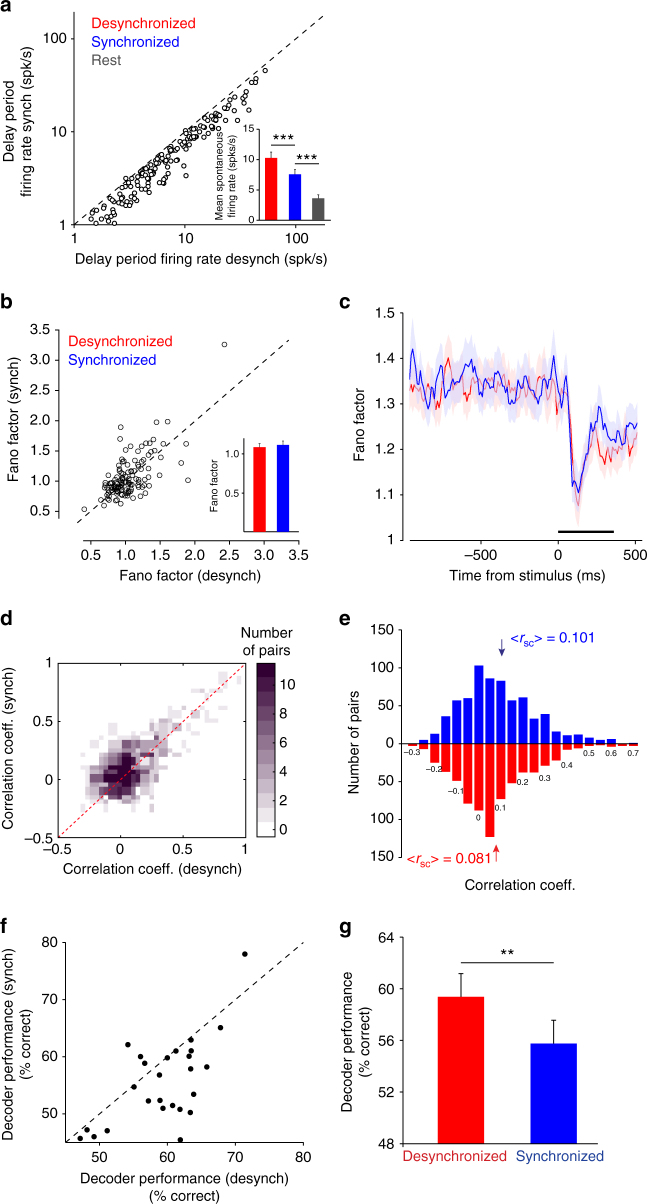
Cortical state impacts the ability of the population of cells to extract sensory information. **a** Firing rates are higher in the desynchronized cortical state. The scatter plot pools data across sessions (each circle represents one neuron). Inset, mean firing rates of neurons in desynchronized and synchronized trials (***P < 0.0001). **b** Fano factor (variance/mean) in the synchronized states plotted as a function of Fano factor in the desynchronized state for the test stimulus presentation. Inset, average Fano factor for each state (*P* > 0.05). **c** Fano factor plotted in a 50 ms sliding window (step size 10 ms) for the 1 s before test stimulus presentation (black bar) to 500 ms past stimulus onset. **d** Three-dimensional histogram of the correlation coefficients plotted for the synchronized and desynchronized states. The *z* axis represents the number of pairs in each correlation coefficient window. The dotted red line represents unity. **e** Histogram of the evoked correlation coefficients for the synchronized and desynchronized trials. The arrows represent the mean <*r*_sc_> for each group. **f** Scatter plot of decoder performance (% correct classification) in the synchronized vs. desynchronized state. Each circle represents a session. **g** Average decoder performance was significantly higher in the desynchronized state (**P < 0.005). Error bars represent standard error

We assessed the impact of fluctuations in cortical state on the neurons’ firing rates and correlated variability, as these variables have been shown to influence the available information in a population of cells^16–18^. While previous studies have shown that anesthesia and sleep increase correlations^9, 11, 15, 19, 20^, whether this measure is influenced by fluctuations in the state of cortical populations during wakefulness is less understood^7^. As shown in Fig. 2a, by comparing the mean firing rates for all the neurons during the delay period, we found a significantly increased firing rate in desynchronized trials (*P* < 0.0001, paired *t*-test, *n* = 176 cells). This difference was preserved when we recalculated the delay-activity firing rate of one neuron while computing the PSI based on all the other simultaneously recorded neurons while excluding the neuron itself (*P* < 0.0003, paired *t*-test, *n* = 176 cells). In contrast, even though we expected pre-stimulus activity to be correlated to stimulus-evoked activity^21, 22^ (measured during the 367 ms presentation of the test stimulus) there was no significant difference in the mean evoked rates between the two states (*P* = 0.903, paired *t*-test, *n* = 176 cells). This difference between the mean evoked firing rates remained statistically nonsignificant when trials were classified as synchronized and desynchronized based on the ongoing activity preceding the first stimulus presented in the task (*P* = 0.3176, paired *t*-test, *n* = 176 cells; Supplementary Fig. 3).

We further examined whether cortical state modulates the relationship between response magnitude and variability of single neurons. There was no significant difference in the neurons’ Fano factor^23–25^ between the synchronized and desynchronized trials (Fig. 2b, *P* = 0.23, paired *t*-test, *n* = 176 cells; see also Supplementary Fig. 4). However, we noticed a stimulus-driven decline in variability for both trials types (Fig. 2c), in agreement with previous work^23^. The lack of change in Fano factor and evoked responses as a function of cortical state argues against fluctuations in attention as a variable that could have been associated with the level of population synchrony. Indeed, we quantitatively compared the effects of cortical state on neuronal responses and Fano factor with those induced by attention by performing additional controls (*n* = 8 sessions) in which V4 responses to attended and unattended stimuli were recorded (*n* = 43 cells, see Methods). However, whereas attention significantly decreased the Fano factor (FF) and increased mean responses of individual neurons^26–28^—mean FF (unattended)=1.29 ± 0.06; FF (attended)=1.17 ± 0.04; *P* = 0.034, Wilcoxon signed rank test and mean response (unattended)=10.2 ± 2.24; mean response (attended)=12.8 ± 1.61; *P* < 0.01, Wilcoxon signed rank test—the corresponding effects associated with cortical state were statistically nonsignificant (mean FF(synch)=1.11 ± 0.05; FF(desynch)=1.09 ± 0.05; *P* > 0.2 for both comparisons).

Previous studies have demonstrated that cortical state can strongly influence pairwise correlations during anesthesia^11, 15^, but little is known about how fluctuations in synchronized population activity during wakefulness impact correlations. We thus examined whether population synchrony impacts trial-by-trial fluctuations in correlated variability, or ‘noise correlations’, in synchronized and desynchronized trials. We first calculated the mean correlation coefficient for all the pairs during the delay period. Confirming our expectation, correlations were higher in the synchronized state (*P* < 0.0001, paired *t*-test, *n* = 703 pairs)^15^. Indeed, whereas correlations were relatively close to zero in the desynchronized state, they significantly increased in the synchronized state. Stimulus-evoked correlations during the test stimulus period (Fig. 2d, e) were significantly higher in the synchronized state (*P* < 0.001, paired *t*-test, *n* = 703 pairs; see also a comparison with correlations during rest, Supplementary Fig. 5). On a session-by-session basis, neuronal correlations during the delay period are strongly correlated with those during stimulus presentation (*r* = 0.706, *P* < 0.00001, Pearson correlation, *n* = 28 sessions). Thus, the state of the population in the delay interval before the test stimulus influences not only correlated variability during the delay interval but also correlations in evoked responses. As revealed by the distribution of correlation coefficients across sessions (Fig. 2e), the mean decrease in correlations from synchronized to desynchronized state was approximately 20%, which is within the range of correlation change observed during attention^26, 27^ and after adaptation^29^.

The state-dependent changes in neuronal responses and correlations raise the possibility that trial fluctuations in cortical state may influence the accuracy with which the population of cells can decode stimulus orientation. We used the neurons’ mean responses to the target and test stimuli in each trial to train an optimal linear classifier to discriminate between these two stimuli, exactly as animals were required to do in the task. Better decoder performance corresponds to an increase in sensory information^30^. To characterize how PSI modulates information, we pooled all the non-match image orientation trials, and then split the data into trials with either high or low population synchrony. Across recording sessions, the network of cells performed significantly better in the desynchronized trials, which were also characterized by lower correlated variability (*P* < 0.005, paired *t*-test, *n* = 26 sessions, two sessions were discarded for insufficient number of training and test trials, Figs. 2f, g). Altogether, these results indicate that trial-by-trial fluctuations in network synchrony significantly influence correlated variability and the amount of sensory information encoded in population activity.

### Cortical state influences behavioral performance

**Fig. 3:**
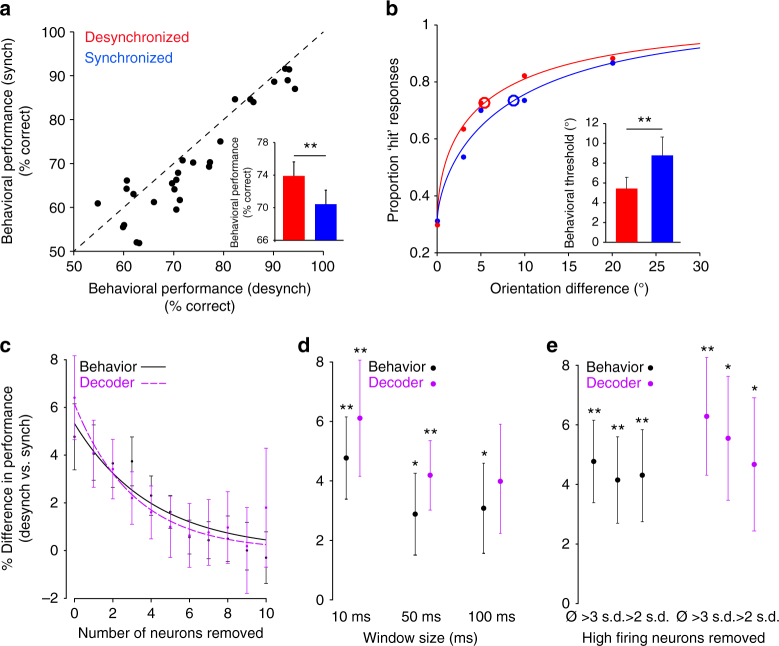
Population synchrony impacts behavioral performance. **a** Scatter plot of the behavioral performance (% correct responses) in synchronized vs. desynchronized trials. Each point represents one session. Inset, mean behavioral performance is significantly higher in desynchronized trials (**P < 0.001). Error bars represent standard error. **b** Psychometric curves associated with the synchronized and desynchronized cortical states. Large circles represent the psychometric thresholds. Solid dots represent the cumulative proportion of hit responses across trials and sessions for each orientation difference. We did not include the 2° orientation difference since it was only used in four sessions (however, the difference in the proportion of hit responses between desynch vs. synch trials was 5.6%). Inset, average behavioral performance threshold is significantly lower in desynchronized trials (**P < 0.005). **c** Percent difference in behavioral and decoder performance between the desynchronized and synchronized trials using the cell-dropping procedure (behavior: black; decoder: purple). The two curves represent exponential fits. Error bars represent standard error. **d** Percent difference in behavioral and decoder performance between desynchronized and synchronized trials for different window sizes to define PSI (**P < 0.005, *P < 0.05). **e** Percent difference in behavioral and decoder performance between desynchronized and synchronized trials after removing the high-firing rate (outlier) neurons from the population. Ø represents no neurons removed, >3 s.d. and >2 s.d. represent removing neurons with average spontaneous firing rates >3 s.d. and 2 s.d. above the population mean (**P < 0.005, *P < 0.05)

Based on our decoder analysis, we hypothesized that the higher encoding accuracy in the desynchronized state may be associated with an increase in behavioral performance in the discrimination task. After splitting the trials (in each session) into low and high population synchrony trials (using our PSI measure), we computed the percentage of correct behavioral responses corresponding to the two cortical states (in the non-match trials). Indeed, behavioral performance was significantly higher in the desynchronized trials (Fig. 3a, b; *P* < 0.001, paired *t*-test, *n* = 28 sessions; see also Supplementary Fig. 6 for individual animal performance). Since this analysis pooled all non-match image orientation trials, we re-examined behavioral performance by computing the orientation discrimination threshold in each session (obtained by fitting the psychometric curves to Weibull functions, see Methods). Confirming the results in Fig. 3a, b, we found that the orientation discrimination threshold was significantly lower in the desynchronized state (Fig. 3b inset, synchronized: 8.76 ± 1.32; desynchronized: 5.41 ± 1.10, *P* < 0.005, paired *t*-test, *n* = 28 sessions). Furthermore, by examining the relationship between decoder and behavioral performance in the synchronized vs. desynchronized cortical states (by pooling the 10° and 20° orientation trials), we found a significant correlation between the two across sessions (*r* = 0.5741, *P* = 0.0022, Pearson correlation, *n* = 26 sessions; Supplementary Fig. 7). This indicates that the greater the improvement in classifier performance in the desynchronized state the greater the improvement in behavioral performance. Indeed, we found even larger differences in behavioral performance between the two network states when comparing the top third, or top quarter, most synchronized and desynchronized trials where the PSI difference between the two groups is larger (*P* < 0.001 for thirds; *P* < 0.005 for quarters; paired t-test, *n* = 28 sessions, Supplementary Fig. 8). This analysis provides additional support for our conclusion that population synchrony impacts behavioral performance.

One potential confound when interpreting our behavioral results is that animals might have discriminated stimuli better in the desynchronized state simply because those trials were associated with less difficult test orientations (our range of orientations was 2–20°). This was not the case—when we compared the average number of trials across sessions associated with each cortical state, we found no significant difference across orientations (Supplementary Fig. 9, *P* > 0.05 paired *t*-test, *n* = 28 sessions), indicating that our results were not due to differences in task difficulty. Furthermore, splitting up trials based on task difficulty—‘difficult’ trials (2–5° rotation) and ‘easy’ trials (10–20° rotation)—did not yield a significant difference between the changes in behavioral performance (desynchronized vs. synchronized) between the two groups (*P* = 0.87, paired *t*-test, *n* = 28 sessions). Together, the results in Fig. 3a–d demonstrate that trial-by-trial fluctuations in population synchrony during wakefulness significantly modulate behavioral performance.

### Control analyses

Our measure of population synchrony (PSI) was based on the spiking activity of all the simultaneously recorded neurons within a session. Thus, we reasoned that the impact of PSI on network accuracy and behavioral performance would diminish if the number of pooled neurons was decreased. One could argue that in this case the PSI measure would not accurately reflect the ‘true’ state of the population of recorded neurons, and hence the division of the data into high and low population synchrony trials would not yield significant results. We addressed this issue by sequentially dropping neurons from the population while recalculating the relative difference in behavioral and decoder performance in the two cortical states. That is, after dropping *n* neurons from the population of recorded cells in a given session, we divided the trials into low/high population synchrony trials based on the PSI calculated from the remaining cells. As expected the difference between the behavioral (and decoder performance) effects associated with the two synchrony states gradually diminished as more neurons were discarded from our sample (Fig. 3c, *P* < 0.05, paired *t*-test, *n* = 28 sessions). For instance, when the number of discarded neurons was relatively large (e.g., eight and above), both the decoder and behavioral performance became independent of cortical state as PSI was calculated from the responses of a small number of neurons. Importantly, observing that behavioral and decoding performance increase significantly with the increase in population size suggests that recording simultaneously from very large ensembles of neurons—e.g., thousands of cells—would provide even more accurate measures of population synchrony, which could help explain an even larger fraction of the perceptual and neuronal variability observed experimentally.

What is the extent to which our results depend on the temporal resolution of the PSI measure used in the analyses? We reasoned that using larger time windows, e.g., 50 or 100 ms as opposed to 10 ms (Figs 1 and 2), would still capture the large fluctuations in population synchrony. Indeed, we found that even larger time windows (>10 ms) yield significant differences in behavioral performance between the synchronized and desynchronized states (Fig. 3d, *P* < 0.05, paired *t*-test, *n* = 28 sessions); decoder performance difference remained statistically significant for the 50 ms bin size (*P* < 0.001, paired *t*-test, *n* = 26 sessions), but not for 100 ms (*P* = 0.078, paired *t*-test, *n* = 26 sessions). The fact that the neural and behavioral effects induced by population synchrony are somewhat diminished when using larger time bins indicates that a low-resolution PSI measure is not the ideal tool with which to explain the variability in neuronal and behavioral performance during wakefulness. Lastly, we investigated whether our main results were being driven by a small subset of high-firing neurons which would dominate the population rate. Even after we removed the high-firing neurons with spontaneous firing rates three, or two, standard deviations above the mean population rate behavioral and decoder performance were significantly improved in the desynchronized state (Fig. 3e, *P* < 0.05, paired *t*-test, *n* = 28 and *n* = 26 sessions, respectively).

### Characterizing cortical state based on local field potentials

**Fig. 4:**
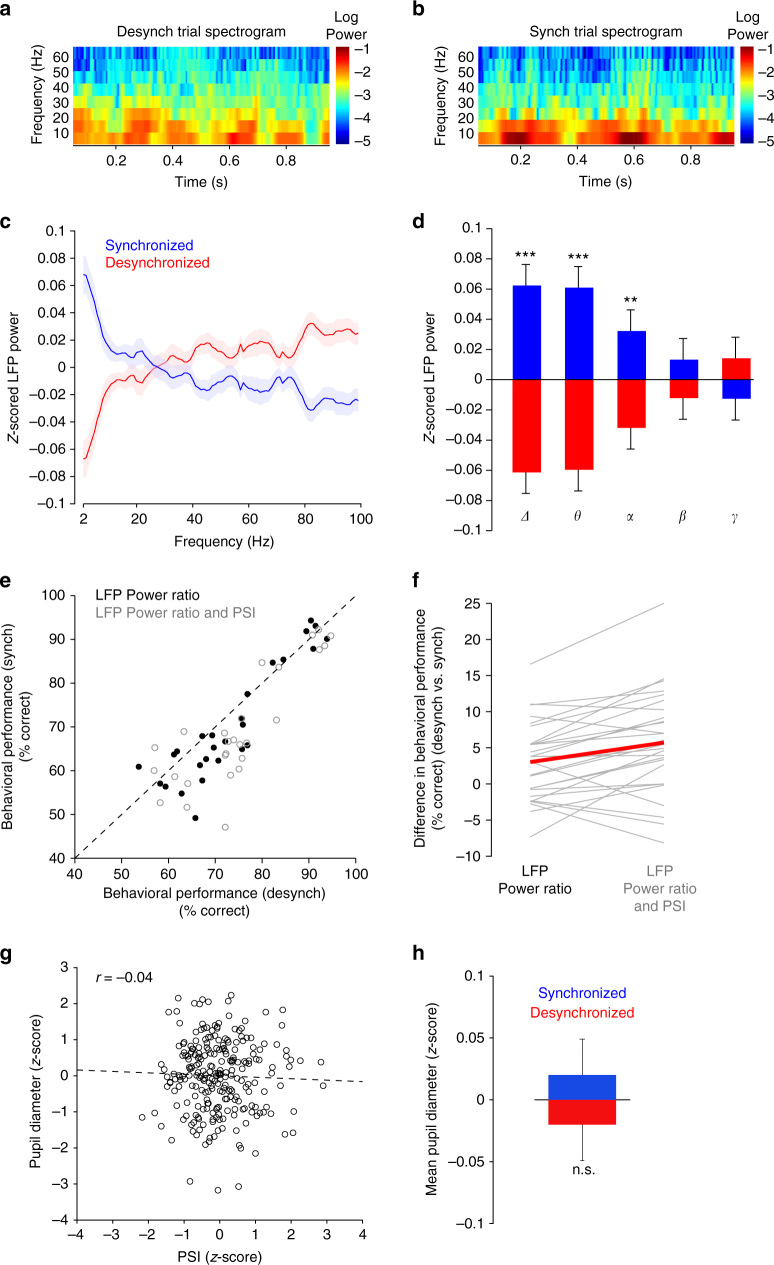
Using LFP power to characterize the functional impact of population synchrony. **a** Example time-frequency spectrogram for a desynchronized trial averaged across all recording electrodes. The power ratio for this trial was 0.799 (P_2–10Hz_/P_10-70Hz_). **b** Example time-frequency spectrogram for a synchronized trial averaged across all recording electrodes. The power ratio for this trial was 3.273 (P_2–10Hz_/P_10-70Hz_). **c** *Z*-scored LFP power in synchronized and desynchronized cortical states (defined by PSI). LFP power was calculated using a 5 Hz rolling window size with a step of 1 Hz. Shaded areas represent standard error. **d** *Z*-scored LFP power in physiologic bands for synchronized and desynchronized trials (as defined by PSI) (*Δ* = 2–4 Hz, *θ* = 4–8 Hz, *α* = 8–12 Hz, *β* = 12–30 Hz, *γ* = 30–100 Hz) (***P < 0.0001, **P < 0.005). **e** Black dots: scatter plot of behavioral performance (% correct responses) in desynchronized/synchronized cortical states (defined by the LFP power ratio). Each point represents one session. Gray circles: scatter plot of behavioral performance in desynchronized/synchronized cortical states. Trials were selected only if they shared the same state of synchrony using both the PSI and the power ratio methods. **f** Difference in average behavioral performance (% correct responses) between synchronized and desynchronized states was higher when cortical state was assessed using both the PSI and power ratio methods (*P < 0.01; **P < 0.0005). The red line connects the mean of each category. **g** Example session showing *z*-scored pupil size as a function of PSI for all trials (all measurements were performed during the delay period). There was no significant correlation between the two variables (*r* = −0.04, *P* = 0.5283, Pearson correlation). **h** Across sessions, the mean pupil diameter in the synchronized state was not significantly different than the mean pupil diameter in the synchronized state (*P* = 0.5124, paired *t*-test)

Although we measured population synchrony based on the spiking activity of single neurons, another way to capture the fluctuations in network activity, ostensibly with less precision, is using local field potentials (LFPs)^4, 11, 31, 32^. In agreement with previous studies, the synchronous state in this alternate classification scheme (Fig. 4a, b) would be associated with high LFP power in the low-frequency range (0.5–10 Hz) and low power in the high-frequency range (10–100 Hz)^7, 11, 31^. Thus, we re-examined the trial-by-trial functional impact of fluctuations in population synchrony by computing the normalized LFP power in the synchronized and desynchronized trials (defined by the PSI measure, Fig. 4c, d and Supplementary Fig. 10). We confirmed our expectation that the synchronous cortical state is associated with an increase in low-frequency LFP power (*P* < 0.0001, paired *t*-test, *n* = 28 sessions) and a decrease in high-frequency LFP power. Importantly, using the LFP power ratio—low-frequency power (2–10 Hz) divided by high-frequency power (10–100 Hz)—as a measure of population synchrony, we found that behavioral performance was higher in the trials characterized by a desynchronized state (*P* < 0.01, paired *t*-test, *n* = 28 sessions, Fig. 4e).

Lastly, we selected the synchronized and desynchronized trials using both the PSI and LFP power ratio criteria. That is, trials were independently classified based on the median PSI and the median power ratio in each session, and only the trials associated with the same state of synchrony in both classification schemes were further analyzed. We found that combining single-unit and LFP signals to define population synchrony led to an improved definition of cortical state—there was an enhanced difference in behavioral performance (*P* < 0.005, paired *t*-test, *n* = 28 sessions, Fig. 4e, f) in the synchronized vs. desynchronized trials relative to the case when power ratio alone or PSI alone were used to measure population synchrony (*P* < 0.05 for both comparisons). These results are consistent with previous findings that fluctuations in LFPs can be used to improve the prediction of the variability of spiking neuronal responses in visual cortex^33, 34^.

### Fluctuations in synchrony and local network activity

Previous studies in rodents have shown that fluctuations in cortical state covary with global arousal, and are correlated with changes in neuronal responses and behavioral performance^1–5^. Although we reasoned that arousal would be expected to cause more persistent, temporally correlated, changes in cortical activity than those reported here^6–8, 11, 12^ (fluctuations in population synchrony that we measured were uncorrelated across trials, Fig. 1j), we nonetheless directly tested whether our fluctuations are related to trial-by-trial changes in global arousal. Several measures of arousal have been proposed in the literature, mainly in humans, such as pupil size, EEG low-frequency power and galvanic skin conductance^35–39^. We examined the correlation between trial-by-trial PSI and pupil size, and between PSI and EEG low-frequency power.

First, we recorded pupil diameter throughout the behavioral task (see Methods) from a subset of sessions exhibiting significant correlations between population synchrony and behavioral performance (*P* < 0.05; Pearson correlation, *n* = 8 sessions, Fig. 4g, h). Previous studies have shown that pupil dilation is associated with desynchronization of neural activity whereas pupil constriction is associated with synchronized neural responses^1–5^. However, there was overall, a statistically nonsignificant relationship between pupil size and PSI (Fig. 4g, *P* > 0.05, Pearson correlation, *n* = 246 trials) in each session. There was also no significant difference in pupil size between the synchronized and desynchronized cortical states across all sessions (Fig. 4h) (*P* = 0.51, paired *t*-test).

Additionally, we recorded scalp EEGs (see Methods) in a subset of the sessions (*n* = 12) for which we found significant correlations between population synchrony and behavioral performance (*P* < 0.01, Pearson correlation). Arousal is associated with decreased low-frequency EEG power^36–38^. We thus examined the relationship between the EEG power in several physiological bands and population synchrony across sessions (Supplementary Fig. 11). Only a small number of sessions exhibited statistically significant relationships (*P* < 0.05, Pearson correlation) between PSI and EEG power in specific frequency bands. Specifically: (i) for the O1 electrode only one out of 12 sessions exhibited a significant correlation between PSI and EEG power in *θ*, *α* and *β* bands, (ii) for the C3 electrode only 1 out of 12 sessions exhibited a significant correlation between PSI and EEG power in *θ* and *α* bands, and two out of 12 sessions exhibited a significant correlation between PSI and EEG power in the *Δ* band, and (iii) for the F3 electrode only one out of 12 sessions exhibited a significant correlation between PSI and EEG power in *θ* and *β* bands, and two out of 12 sessions exhibited a significant correlation between PSI and EEG power in the *α* band. However, across sessions and electrodes we did not find a statistically significant difference between the two groups of PSI values in any frequency band (*P* > 0.1, paired *t*-test). Taken together, these results indicate that trial fluctuations in arousal, as measured by pupil size and EEG power, are not correlated with the fluctuations in population activity measured by our experiments. Therefore, the trial-by-trial dynamics of population synchrony revealed here reflect the dynamics of local population activity rather than global cortical states controlled by arousal.

## Discussion

Cortical states have been traditionally examined using EEG techniques, and only relatively recently using single-cell electrophysiology. Multiple electrodes and intracellular studies have allowed the study of patterns of population responses and their spontaneous fluctuations. Previous studies aimed at understanding cortical states at the single-cell level have revealed the existence of a synchronized state during slow-wave sleep and a desynchronized state during wakefulness^40, 41^. However, although it has been acknowledged that fluctuations in ongoing activity during wakefulness change the state of neuronal populations involved in stimulus processing^13^, whether and how cortical states interact with incoming stimuli to influence visual coding, and subsequently perceptual performance, has remained poorly understood.

We found that rapid fluctuations in population synchrony during wakefulness impact both the information encoded in network activity and behavioral performance. That is, synchronous fluctuations in population activity influence the strength of neuronal responses and noise correlations such as to decrease network discrimination performance and reduce perceptual accuracy even when the animal is seemingly alert and actively engaged in the task. The functional impact of cortical state has been previously examined in anesthetized animals^11, 14, 15^ and in awake mice, using measures of arousal such as pupil diameter^7, 8^. However, whether fluctuations in cortical population synchrony influence both the accuracy of sensory discriminations and behavioral performance has remained mysterious. Importantly, we demonstrated that the fluctuations in population synchrony revealed here reflect local changes in network activity rather than global cortical state dynamics induced by general arousal.

An important way in which cortical states can impact the capacity of neuronal populations to encode information is by influencing correlations between neurons. Indeed, the structure of correlations has been shown to influence the available information in the responses of a population of cells^16–18, 29, 42^ and possibly limit behavioral performance^16, 43^. Recently, pairwise correlations have been found to be strongly influenced by global fluctuations in the population response across different wakeful brain states^4, 6, 7, 44^. In rat visual cortex, correlations have been shown to increase from wakefulness to anesthesia^9^. In anesthetized rat auditory cortex, correlations were almost zero when the neuronal population was in a desynchronized state^14^, whereas in monkey V1, correlations varied depending on cortical state between anesthesia (strong correlations) and wakefulness (weak correlations^11, 28^). In contrast, our results reveal noise correlation values that are consistent with previous reports in behaving monkey V4^27, 45^—although correlations were decreased when neurons are desynchronized, they remained relatively strong (around 0.08). Thus, our results are inconsistent with studies proposing that during wakefulness, noise correlations are reduced to values close to zero when the population of neurons operates outside of the synchronous state^11, 14, 28^.

Most work on synchronized and desynchronized cortical states has been performed in anesthetized rodents where it has been reported that varying the depth of anesthesia strongly influences ongoing and evoked firing rates^9, 20, 46, 47^. Recent work in mouse V1 has explored the impact of cortical state on neuronal responses and behavior^7, 12^. However, there are important differences between our study and earlier investigations. In addition to the difference in species (monkey vs. mouse), our definition of cortical state is restricted to the local population activity that is monitored while the animal performs the task. In contrast, previous investigations measured global fluctuations in behavioral state, such as arousal and their impact of neuronal responses. For instance, it was reported that rapid variations in locomotion and arousal (as measured by pupil diameter) control sensory-evoked responses and spontaneous activity of individual neurons^7, 48^, and that noise correlations were lower during locomotion than quiescence, while evoked responses were stronger^7, 48^. Global state fluctuations, such as arousal, were also found to modulate the membrane potentials of auditory cortical neurons in mice trained on a tone-in-noise detection task^8^. Importantly, arousal was found to modulate behavioral performance by enhancing sensory-evoked cortical responses and reducing background synaptic activity. However, previous studies of global cortical states did not investigate how the fluctuations in population synchrony jointly influence the information encoded by ensembles of neurons and behavioral performance.

One apparently surprising finding is that the fluctuations in cortical state are accompanied by marked changes in neuronal correlations, but no changes in evoked responses. Nonetheless, this is consistent with recent work performed in awake animals demonstrating unchanged evoked firing rates as animals moved from quiescence to alert behavioral states (i.e., synchronized to desynchronized)^7, 49^. In contrast, increased evoked rates have been reported as animals transitioned from quiescence to locomotion or during active whisking^1, 4, 50^. However, a recent study^7^ disambiguated arousal from locomotion to find that arousal, by itself, did not significantly influence evoked rates, although it did influence correlated variability, in agreement with our findings. The decrease in correlations in the desynchronized state could be explained by an increase in local inhibition, which is consistent with previously reported increases in inhibitory responses in visual cortex during wakefulness^51^. Indeed, the balance between excitation and inhibition has been previously shown to regulate correlated variability, and stronger inhibition has been related to decreased correlations^14, 18, 52^. Further, we suggest that a similar mechanism (changes in the balance between local excitation and inhibition) could explain the lack of change in evoked responses in the two cortical states.

One of our important results is that the observed trial fluctuations in population activity have a local circuit origin rather than reflecting fluctuations in global brain state, possibly due to general arousal or attention. There are two main reasons that led to this conclusion. First, our experiments failed to find a significant link between population synchrony and arousal, as measured by pupil size and EEG power. This could be explained by the fact that our task demands might not have elicited large fluctuations in arousal in a way that could influence population synchrony. That is, in every session, monkeys were well rested and consistently performed at high levels. The trials in which discriminations were easy or difficult were not cued and correct responses ended up being rewarded with the same amount of juice. In contrast, previous studies reporting a relationship between cortical state and arousal were performed while animals underwent large changes in behavioral state as they transitioned from quiescence to locomotion or active whisking^1, 4, 6–8, 12^.

Second, our analysis of neural data has ruled out that the fluctuations in V4 population activity are related to changes in attention. Although some of the changes in neuronal responses observed here, such as changes in noise correlations and LFP power ratio (Figs. 2d, e and 4a–d) may be consistent with diminished attention^26, 27^ in the synchronized state, our findings that the magnitude of evoked responses and the Fano factor do not change as a function of cortical state argue that attention is not the source of fluctuations in local population activity. To further examine the role of attention, we measured the relationship between the LFP gamma power, one of the hallmarks of attention^53–55^, and PSI by pooling all the trials across sessions—we found a mean correlation value of *r* = −0.033 ± 0.024 (*P* > 0.1; only 5 out of 24 sessions were associated with statistically significant correlation values). Finally, since attention has been found to increase the bursting rate of neurons, we computed the percentage of bursting spikes (spikes with interspike intervals ≤5 ms^56, 57^) on individual trials in both the synchronized and desynchronized states. However, there was no significant difference in bursting between the two cortical state conditions across sessions (*P* = 0.81, paired *t*-test, *n* = 28 sessions, Supplementary Fig. 12). Altogether, these analyses indicate that attention is highly unlikely to contribute to the fluctuations in population synchrony examined here.

What type of mechanism could explain the fluctuations in local population activity during wakefulness? We propose that while the animal is engaged in the task cortical networks spontaneously fluctuate between different degrees of synchrony. That is, the observed dynamics of population synchrony represent an emergent property of local V4 circuits. In support of this mechanism, it has been previously suggested^13^ that a spontaneous, transient, increase in recurrent synaptic activity^58, 59^ could cause an increase in cortical responses (UP state), and subsequent synaptic depression^60, 61^, or after-hyperpolarizing potassium conductances^58^ could reduce network excitability (DOWN state). This mechanism is supported by computational models—for instance, it has been shown^62^ that recurrent models relying on strong excitatory connections and depressing synapses can spontaneously transit between UP and DOWN states of different durations controlled by intrinsic local noise amplitude. Other local network mechanisms for synchronized and desynchronized cortical states have been proposed which rely on spike-timing-dependent plasticity^63^ and integrate-and-fire models of excitatory and inhibitory neurons with nonlinear membrane currents^64^.

A recent study in area V4 of behaving monkey^65^ using linear arrays has reported striking ON and OFF state transitions during wakefulness (both during passive fixation and attention). That is, neurons within a cortical column were highly synchronized throughout trials as they rapidly fired together for hundreds of ms, and then responded at a much slower rate (see related studies in area V1 of anesthetized and fixating macaque^22, 66^); the momentary phase of the local ensemble activity predicted behavioral performance in an attentional task. There are several major differences between our study and Engel et al.^65^. First, the cortical state definition is fundamentally different between the two studies. Instead of focusing on rapid ON/OFF firing rate fluctuations (within 100 ms) before and during stimulus presentation, we captured the state of a network based on the degree of oscillatory activity measured over a 10-fold larger time interval. Thus, our definition of state—synchronized or desynchronized population activity—represents the classical definition of ‘state’ used in previous studies examining brain activity during wakefulness, anesthesia, drowsiness or sleep. Cortical state measures based on the strength of the population response over a short time interval make it impossible to distinguish between ‘high’ or ‘low’ response states occurring when the population of cells is synchronized vs. isolated state transitions occurring when the population is desynchronized. This is an important limitation since cortical networks have been shown to primarily operate in a desynchronized regime during wakefulness. Second, Engel et al.^65^ found that the relative duration of their response states is modulated by arousal and attention. Therefore, unlike the cortical states described in our study that have a local circuit origin, Engel et al.^65^ have captured state transitions related to global fluctuations. Third, unlike our study, Engel et al.^65^ have shown that ON/OFF state transitions occur continuously across trials regardless of behavioral state, which implies that cortical columns always operate in a synchronized mode. However, such highly synchronized neuronal activity within a cortical column would lead to high correlated variability beyond previously reported values in V1 and V4, and regardless of cortical layer^18, 67, 68^. Finally, Engel et al.^65^ did not decode the network response to measure how cortical state influences the relationship between the information encoded in population activity and behavioral performance; examining this relationship is the central goal of our study.

To further relate our study with Engel et al.,^65^ we examined the relationship between the ON/OFF population response states and behavioral performance. Specifically, since the striking ON/OFF state transitions across trials were defined by a tight temporal alignment of MUA responses^65^, we exclusively focused on our ‘synchronized’ trials in each session. For those trials, we examined the relationship between neuronal responses before stimulus presentation (ON/OFF, i.e., higher or lower than median pre-stimulus population response) and behavioral performance by selecting pre-stimulus intervals of either 200 or 100 ms. However, we failed to find a statistically significant relationship between behavioral performance and ON/OFF states: for 200 ms—behavioral performance (OFF) = 69.5 ± 2.8%; BP(ON) = 69.0 ± 2.7% (*P* = 0.68, paired *t*-test); for 100 ms—BP(OFF) = 70.1 ± 2.5%; BP(ON) = 68.6 ± 2.6% (*P* = 0.33, paired *t*-test). These results represent further evidence that behavioral performance is impacted by cortical state captured by the oscillatory population activity before stimulus presentation rather than the response magnitude of neurons before stimulus presentation.

Our measure of population synchrony (PSI) is defined as standard deviation divided by the mean of the population spike count, and hence it depends on mean firing rate. Although we used another measure of cortical state based on LFPs to confirm its influence on sensory coding and behavior, we further classified trials based on the standard deviation of the population rate (the numerator in the PSI equation) without normalizing by the mean firing rate. That is, trials associated with a high standard deviation of the population rate were considered ‘synchronized’ trials and trials associated with a low standard deviation of the population rate were considered ‘desynchronized’ trials. However, even in this case we found a significant difference between behavioral performance in the synchronized vs. desynchronized trials (*P* = 0.0015). Additionally, we extended our analysis by computing a discrete Fourier transform of the population rate in each trial, and sorted trials based on the power of the Fourier coefficients between 2 and 10 Hz (high-power trials were classified as synchronized, and low-power trials were classified as desynchronized). Using this new metric of population synchrony we still found a significant difference between behavioral performance in synchronized and desynchronized trials (*P* = 0.0079, paired *t*-test, *n* = 28 sessions). We also found a high trial-by-trial correlation between PSI and Fourier low-frequency power of the population rate (*r* = 0.7190, *P* < 10^−26^, Pearson correlation; >75% of trials were overlapped). Furthermore, splitting trials into low and high PSI groups and comparing the low-frequency power based on Fourier coefficients revealed that low-frequency Fourier power was significantly different between the two PSI groups (*P* < 10^−8^, paired *t*-test). These new controls further support that our main results are not dependent on the changes in mean firing rates between the two cortical states.

It was recently shown that slow global fluctuations in population activity do not target neurons based on whether or not they are task informative^69^. Although we did not detect a slow global fluctuation in our data, we nonetheless divided our cells (separately for each session) into two groups depending on their stimulus sensitivity (mean *d′*, averaged across all orientation differences), and for each group we split the trials into synchronized and desynchronized (based on the corresponding PSI values). Subsequently, we computed behavioral performance (BP) associated with each group of trials. The results—BP(high *d′*, desynch) = 73.34% ± 0.02%; BP(low *d′*, desynch) = 73.58% ± 0.02%; BP(high *d′*, synch) = 70.98% ± 0.02%; BP(low *d′*, synch) = 70.80% ± 0.02%—reveal that the difference in behavioral performance between the synchronized and desynchronized trials is not statistically different between the low and high *d′* groups of neurons (*P* > 0.1, Wilcoxon signed rank test). These results support the fact that fluctuations in population synchrony modulate behavioral performance regardless of the task involvement of the participating neurons.

In principle, synchronous fluctuations in cortical neurons could impact the encoded information and perceptual decisions only if downstream brain regions do not have access to the fluctuating gain factor caused by synchronous fluctuations^30, 70^. In contrast, if downstream areas, such as lateral intraparietal area or medial superior temporal sulcus, are involved in generating synchronous fluctuations in V4, a downstream decoder could simply divide out the fluctuating gain^70^, recover the full input information, and hence behavioral responses would likely not be impacted in any way. However, our results support the former hypothesis, in agreement with our conclusion that fluctuations in population synchrony are locally generated within intrinsic circuits.

Altogether, our results demonstrate that the structure of variability in local cortical populations is not noise but rather controls how sensory information is optimally integrated with ongoing processes to guide network coding and behavior. However, to further elucidate the functional role of fluctuations in local population synchrony, future studies are needed to causally manipulate the state of the cortex and measure its impact on the trial-by-trial population code and behavioral accuracy. Importantly, it remains to be seen whether rapid fluctuations in population responses during wakefulness are coordinated across brain areas and whether the multi-area spatiotemporal pattern of population activity is relevant for behavior. Future research will also elucidate whether the relationship between fluctuations in neuronal responses, network coding, and behavioral performance is restricted to sensory areas or whether it is a component of a more general coding strategy in downstream areas.

## Methods

### Surgical procedures

All experiments were performed in accordance with protocols approved by the US National Institutes of Health Guidelines for the Care and Use of Animals for Experimental Procedures and were approved by the Institutional Animal Care and Use Committee at the University of Texas Health Science Center at Houston.

A titanium head post was implanted in medial frontal region with the help of multiple anchor screws. Following a recovery period of about 10 days, monkeys were trained for 3–4 months on visual fixation and discrimination tasks. After the monkey learned the tasks, a recording chamber (inner diameter of 17 mm) for single-unit multiple electrode recording was cemented over area V4 (according to magnetic resonance imaging map). A few stainless steel screws were inserted into the skull around the recording chamber and a thin stainless steel wire was wrapped around the screws for additional support.

### Behavioral task

Two adult male rhesus monkeys (*Macaca mulatta*), age 7 and 12 years old, were trained in a delayed-match-to-sample task in which they had to indicate whether two successively presented natural images had the same or different orientation (Fig. 1a; *n* = 28 sessions). After monkeys fixated for 400 ms, a target stimulus was flashed for 367 ms, and after a delay period of 1250 ms, a test stimulus flashed for 367 ms. In approximately half of the trials, the test stimulus had the same orientation as that of the target (‘match’ condition). In the other half of the trials, the test orientation was rotated from the target by 2°, 3°, 5°, 10° or 20° (‘non-match’ condition). Animals were trained to release a bar on match trials and hold the bar on non-match trials in order to receive a juice reward. Match and non-match trials were randomly interleaved (we collected at least 500 trials in each session). The inter-trial interval was 10 s.

To calibrate our measure of population synchrony, in a subset of sessions (*n* = 6) we allowed the animals to rest quietly in the dark for 20–30 min following the behavioral task, while electrophysiological recordings continued. During this rest period, a white background noise was played on a speaker to prevent external sounds from arousing the animals. We monitored eye position using the eye tracker and night vision video monitoring. The timing of rest was carefully controlled such that monkeys began the rest at approximately 14:00 hours, which is around the time when monkeys naturally take daytime naps^71^.

In the attention control experiments (*n* = 8 sessions), the animals performed a delayed match-to-sample task with two modes: ‘attention in’ and ‘attention out’ conditions, which were cued by 5–10 orientation discrimination or color detection trials run before the beginning of each block of attention/inattention trials. Only one spatial location was behaviorally relevant on a given trial. One location was inside the receptive fields of the neurons being recorded (we used Crist grid arrays) and the other location was outside the receptive fields, diametrically opposed at the same eccentricity. When the animal’s attention was directed to the stimuli in the receptive fields of the neurons being recorded, the neurons’ responses were putatively relevant to the task the animal was performing and we refer to that condition as ‘attention in’. When the animal’s attention was directed to the colored stimuli outside the receptive field of the neuron being recorded, its responses were putatively irrelevant to the animal’s task and we refer to that condition as ‘attention out’. The attention ‘in’ and ‘out’ trials were grouped in blocks of 100 trials. The orientation discrimination task was identical to that performed in our basic experiment (Fig. 1a). The stimuli outside the receptive field were 5° red/green colored square patches with a two-dimensional Gaussian profiles (isoluminant on a gray background) each presented for 367 ms (the delay period was 1,250 ms). We used an equal number of trials (200) in each attention condition.

### Visual stimuli

Gray-scale natural stimuli (e.g., deer eating grass, water buffalo, jungle scenes) were generated with Psychophysics Toolbox using MATLAB and presented on a 19” CRT color video monitor (Dell, 60 Hz refresh rate). Only one image was presented in each session. Stimuli ranged in size from 8–10°, and were presented at 3–10° of eccentricity. Stimulus location and size were optimized in each session such as to stimulate the largest number of simultaneously recorded cells. Stimulus presentation was recorded and synchronized with the neural data using the Experiment Control Module programmable device (ECM, FHC Inc.).

### Behavioral threshold

We divided the trials in two halves based on the median PSI and then fitted the psychometric curve of the behavioral response for the synchronized and desynchronized trials in each session to a Weibull function in order to obtain the discrimination threshold^72^. The Weibull function was normalized by the false alarm rate, *P*_weib_(0). We implemented the following equations:

$${P_{\rm weib}}( {\Delta \theta }) = 1\! -( {1\!\!-\!{P_{\rm weib}}( 0)}) \cdot {k^{{{\left( {\frac{{\Delta \theta }}{\tau }} \right)}^b}}},$$

$$k = \frac{{1\!-\! {P_{\rm weib}}(\tau )}}{{1\!-\!{P_{\rm weib}}(0)}},$$

$$d \prime \left( {\Delta \theta } \right) = z\left( {\Delta \theta } \right)\!-\!z\left( 0 \right) \Rightarrow z\left( \tau \right) = 1 + z(0),$$

$${P_{\rm weib}}\left( \tau \right) = {z^{ - 1}}\left[ {z(\tau )} \right],$$

where *τ* is the discrimination threshold, *P*_weib_(*τ*) is the proportion of correct responses corresponding to *d′* = 1, *z* is the *z*-transform, and *b* is the slope of the Weibull function.

### Eye movement control

On each trial, monkeys were required to fixate on a central point (0.4° in size) within a 2° fixation window. To ensure fixation, eye position was continuously monitored by using an eye tracker system operating at 1 kHz (EyeLink II; SR Research). Eye position was calibrated at the beginning of each experiment with a five-point calibration procedure. We examined whether states of population synchrony were associated with changes in the quality of fixation by measuring the mean eye position, standard deviation of eye position and eye movement velocity along the horizontal and vertical axes during the delay period. The eye-tracker gains were adjusted such as to be linear for the horizontal and vertical eye deflections. The fixation pattern was carefully analyzed offline. Microsaccades were analyzed every 10 ms by using a vector velocity threshold of 10°/s (this corresponds to a 0.1° eye movement between consecutive 10 ms intervals). If a detected microsaccade exceeded 0.25° (fixation instability) the trial was automatically aborted. By analyzing the amplitude of microsaccades, we found that eye movements were not statistically different between the synchronized and desynchronized cortical states (*P* > 0.1). We also calculated the correlation between the timing of synchronized bursts and the timing of eye movements but found no significant relationship in any of our recorded sessions (*r* = 0.002 ± 0.002; *P* > 0.05, Pearson correlation). Pupil size was measured monocularly at 1 kHz sampling frequency (EyeLink II; SR Research). We included in the analysis only the time periods in which luminance was constant and the fixation point was the only stimulus displayed on the screen (during the delay period). For the analysis, pupil size was downsampled at 100 Hz and the median value was taken over the entire delay period in each trial.

### Electrophysiological recordings

We used two types of electrode systems in each monkey: (i) Arrays of parylene-C coated tungsten microelectrodes (MPI, 1–2 MΩ at 1 kHz) grouped in pairs and attached to several micro-drives (Crist) fixed on a grid, and penetrated transduraly through stainless steel guide tubes into the cortex; (ii) 16-channel linear arrays (Plexon) with contacts spacing at 100 μm advanced using the NAN drive system (Plexon) attached to the recording chamber. In each session, we advanced up to 8 tungsten microelectrodes and/or 2 linear arrays into area V4. We recorded up to 17 units simultaneously in each session. The cells were recorded parafoveally (2–8° eccentricity) and had receptive fields (RFs) within 2° of each other (center-to-center). Both the target and test stimuli fully covered the cells’ RFs. Cells unresponsive to the visual stimuli (*P* > 0.05) were excluded from the analysis.

Real-time neuronal signals were processed with the Multichannel Acquisition Processor system (MAP, Plexon Inc.) at a sampling rate of 40 kHz. The signals were first filtered by a preamplifier box into spike channels (150 Hz–8 kHz, one pole low-cut, three pole high-cut, with programmable referencing, 50× gain) and field potential channels (0.07, 0.7, 3–170, 300, 500 Hz user selectable, one pole low-cut, one pole high-cut, 50×). Single-unit signals were further amplified, filtered, and viewed on an oscilloscope, and heard through a speaker. The spike waveforms above threshold were saved and fine sorted after data acquisition was terminated using Plexon’s offline sorter program. After a unit was isolated, its receptive field was mapped with dynamic gratings or using reverse correlation while the animal maintained fixation. Waveforms that crossed a pre-defined threshold (~4 s.d. above the amplitude of the noise signal) were stored for offline analyses. Spike waveforms were manually processed with Plexon’s offline sorter program using waveform clustering parameters such as principle component analysis, along with spike amplitude, timing, width, valley, and peak. Single units were subsequently analyzed using custom scripts in MATLAB.

Raw LFPs were collected at 1 kHz sampling frequency and a second-order notch filter was implemented to remove the 60 Hz line noise. The LFP power was then computed for each recording channel independently using the MATLAB R2015b function bandpower, which calculates the average power via a rectangle approximation of the integral of the power spectral density estimate. The power was then *z*-scored across trials. The mean *z*-scored power was taken across channels for each band in the synchronized and desynchronized trials such that the mean of the combined groups should always be zero. The smallest frequency used to compute LFP power was 2 Hz due to the 1 s inter-stimulus window limitation. A rolling window band of 5 Hz was moved in 1 Hz steps to generate Fig. 4c. Physiological bands were used to construct Fig. 4d (*Δ* = 2–4 Hz, *θ* = 4–8 Hz, *α* = 8–12 Hz, *β* = 12–30 Hz, *γ* = 30–100 Hz). Power ratio was computed as the LFP power in the low band (2–10 Hz) divided by the LFP power in the high band (10–100 Hz). Trials were split into two groups based on the median power ratio. One session was removed from the analysis due to corrupted LFP signal.

### Population synchrony index

The PSI was calculated in each trial by using the coefficient of variation of the population spike count across 100 windows of *T* = 10 ms^14, 15^:

$${\rm PSI} = {\rm Cv} = \frac{{{\sigma _{\rm pop\;{\rm sc}}}}}{{{\mu _{\rm pop\;{\rm sc}}}}}.$$

The analysis period constituted one second of data during the delay preceding the test stimulus presentation (Fig. 1). For analyses pertaining to Figs 2 and 3, we separated all trials into two groups, synchronized and desynchronized, depending on whether the PSI in the delay period was above or below the median PSI across all trials. In Fig. 3d, we recomputed PSI for the delay period using 20 windows of 50 ms duration, and 10 windows of 100 ms duration. We then calculated behavioral and decoder performance in the newly defined synchronized and desynchronized trials for each separate window size.

### Fano factor

To measure firing rate variability, we computed the Fano Factor (FF) for each neuron, defined as the spike count variance divided by the spike count mean. Spike counts were summed in a 50 ms window starting at the onset of the test stimulus (plus a delay of 70 ms to account for the latency of V4 neurons). For each neuron, FF was calculated for each test stimulus separately and then averaged across conditions. In a separate analysis, we slid the 50 ms window in 10 ms steps across the duration of the trial for comparisons with previous work^23^.

### Noise correlations

Correlated variability was computed as described previously^18, 29^ for each pair of neurons using the Pearson correlation coefficient *R*(*x*, *y*) given by:

$$R\left( {x,\ y} \right) = \frac{{\mathop {\sum }\nolimits_{n = 1}^N \left[ {x(n)\!-\!\bar x} \right]\left( {y(n)\!-\!\bar y} \right]}}{{{\sigma _x}{\sigma _y}}},$$

where *N* is the number of trials, $\bar x$ and $\bar y$ are the means of *x* and *y*, respectively, and *σ*_*x*_ and *σ*_*y*_ are the standard deviations of *x* and *y*, respectively^18, 73^.

### Linear classifier

We decoded the neural activity by implementing a linear classifier which fits a multivariate normal density with a pooled estimate of covariance using the MATLAB function, classify. 50% of the data was used to train the classifier and determine an optimal decision boundary. The accuracy of the decoder was then evaluated on the remaining testing set of data. This process was repeated 100 times and classification performance was averaged across repeats. Data was analyzed separately for the synchronized and desynchronized trials of each session. Two sessions were discarded from this analysis due to insufficient number of trials for training and test sets.

### Cell dropping procedure

For each session, we sequentially removed neurons from the population synchrony analysis and then recomputed the behavioral and decoder performance using all combinations of the remaining neurons. For example, in a population of 10 neurons, we started by removing one neuron. Then, we recomputed the % difference in behavioral and decoder performance for the desynchronized and synchronized trials assessed after computing PSI based on the remaining nine neurons. We repeated this procedure for all combinations of nine neurons and then averaged the results across samples. In the case of nine neurons we would have *N* = 10 different combinations. The number of combinations is calculated using the formula: *v*!/*k*!(*v*-*k*)!, where *k* is the combination size and *v* is the number of total neurons in the pool. We next removed two neurons from the total pool of 10 neurons and computed the % difference in behavioral and decoder performance for the desynchronized and synchronized trials assessed after computing PSI based on the remaining eight neurons. In this case, we would have 45 different combinations of eight neurons. This was repeated until we removed all but one neuron from the total population. The final results were averaged across sessions and plotted based on the number of neurons removed from the population.

### EEG analysis

A custom cap composed of EEG, electrooculogram (EOG) and electromyogram (EMG) electrodes was implemented. Specifically, 6 mm cast silver, gold-plated, cup electrodes (Grass Technologies) were attached to an elastic cap fitted to each monkey over the international standard 10–20 system of EEG sites corresponding to F3 (dorsolateral prefrontal cortex), C3 (somatosensory cortex) and O1 (occipital cortex), cf. Berry et al. (2013). Additional electrodes were secured to straps that attached to the cap, and positioned electrodes above the right eye and below the left eye to detect eye movements. An electrode located on the mentalis muscle was used to detect muscle tone. One ear clip electrode was placed on each ear lobe (RE, right ear, and LE, left ear) and all EEG electrodes were referenced to RE and grounded to LE (Berry et al., 2013). EOGs were referenced to LE and grounded to RE during recordings. The electrodes were applied using Ten20 conductive paste. The recorded data was sampled at 500 Hz for all recording sites. EEGs and EOGs were low-pass filtered online at 150 Hz, and EMGs were band-passed filtered between 10 and 250 Hz.

### Statistics

A Kolmogorov–Smirnov test was used to test the null hypothesis that the samples are normally distributed (*P* > 0.05). When the data was normally distributed, a two-tailed, paired *t*-test was used throughout the analysis. Otherwise, when the data was not normally distributed, non-parametric tests were used, as specified in the main text (e.g., Wilcoxon rank-sum for Fig. 1i).

### Data availability

The data that support the findings of this study are available from the corresponding author on reasonable request.

## Electronic supplementary material

Supplementary Information
