# Supplementary material for: Sensory coding accuracy and perceptual performance are improved during the desynchronized cortical state
Source: Nat Commun. 2017 Nov 3;8:1308. doi: 10.1038/s41467-017-01030-4 (PMC5670198; doi:10.1038/s41467-017-01030-4)
Supplement: Supplementary file 1 — Supplementary Information [file 41467_2017_1030_MOESM1_ESM.pdf]

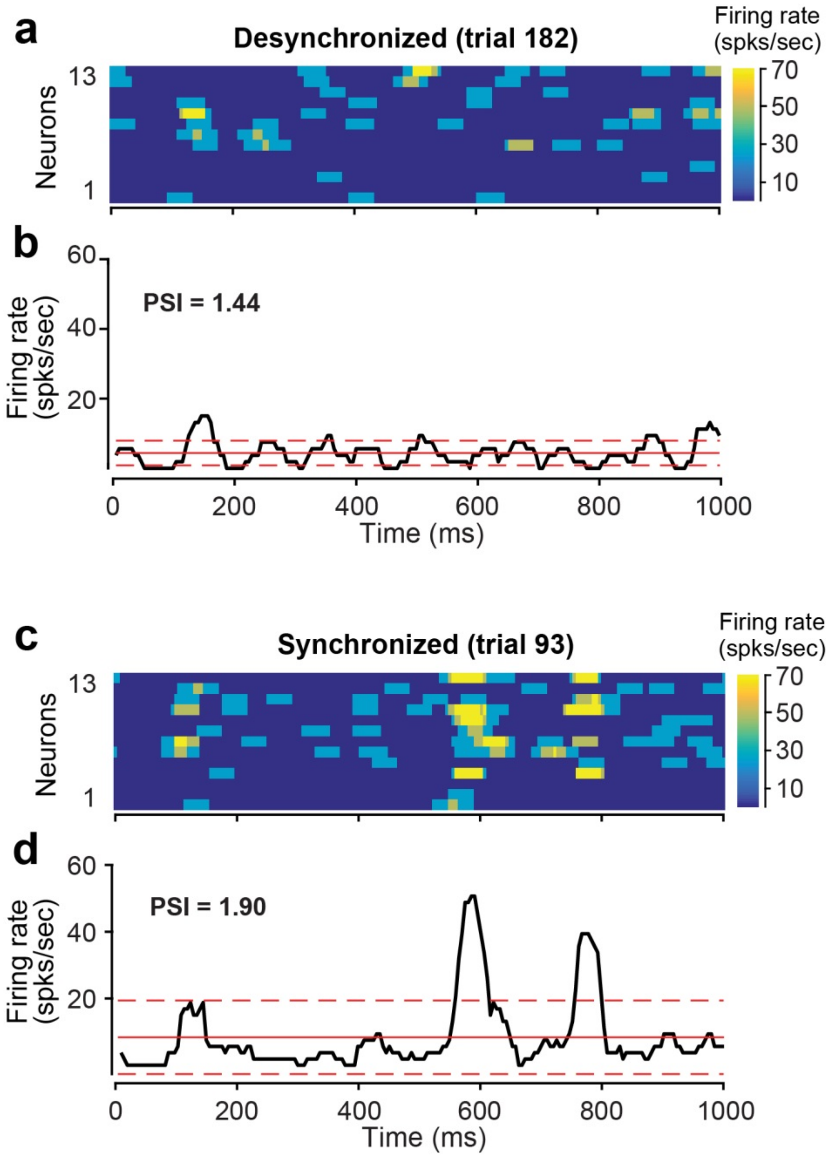

Supplementary Figure 1: Additional examples of population activity in desynchronized and synchronized trials. (a) One example desynchronized trial with 13 simultaneously recorded neurons. The heat map represents the population firing rate for each neuron (40 ms rolling window, 5 ms step). **(b)** The average population firing rate plotted as a function of time for the trial displayed in panel A. Population synchrony index (PSI, see Methods) is denoted. The solid red line indicates the population mean firing rate for the trial, and the red dotted lines indicate 1 standard deviation from this mean. **(c)** One example synchronized trial with 13 simultaneously recorded neurons. **(d)** The average population rate plotted as a function of time for the trial displayed in A. Two periods of synchronized firing can be seen in this trial.

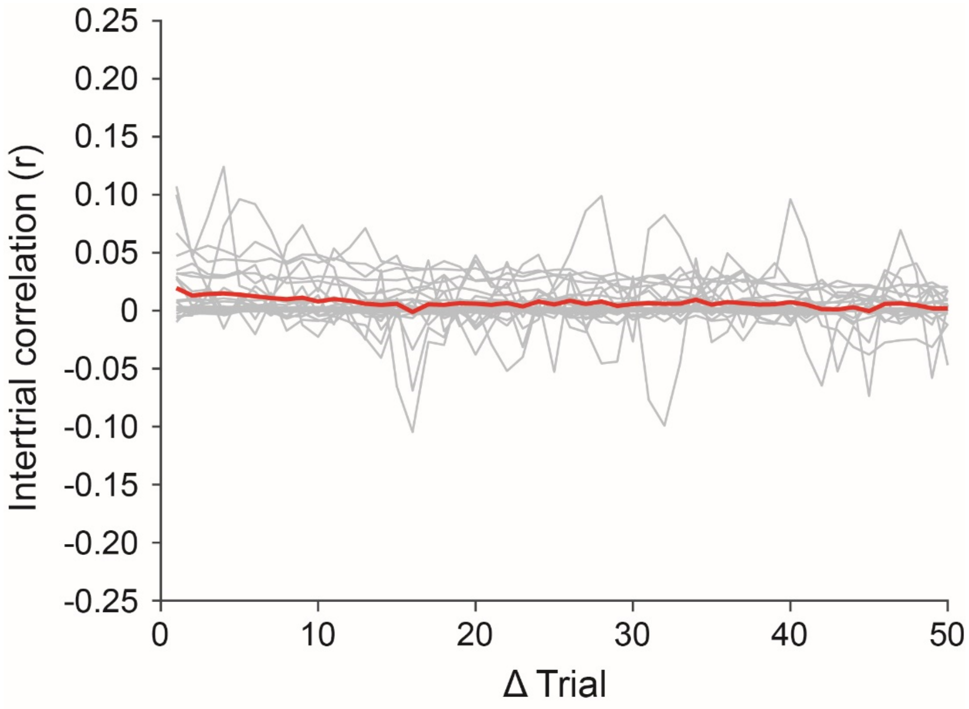

Supplementary Figure 2: Intertrial correlations of trial-by-trial PSI for all sessions. The red trace indicates the mean autocorrelation (across sessions) of trial-by-trial PSI.

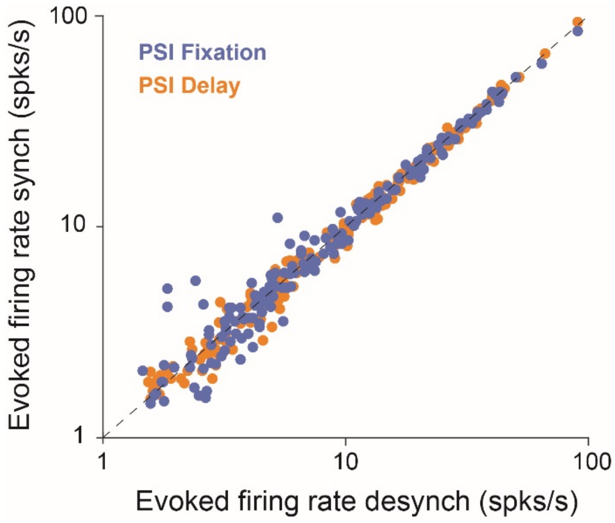

Supplementary Figure 3: Evoked firing rates in the desynchronized and synchronized cortical states defined based on the PSI calculated during the fixation and delay periods. There was no significant difference between the evoked rates using the fixation period to define population synchrony (P = 0.3176, paired t-test) or using the delay period to define population synchrony (P = 0.903, paired t-test).

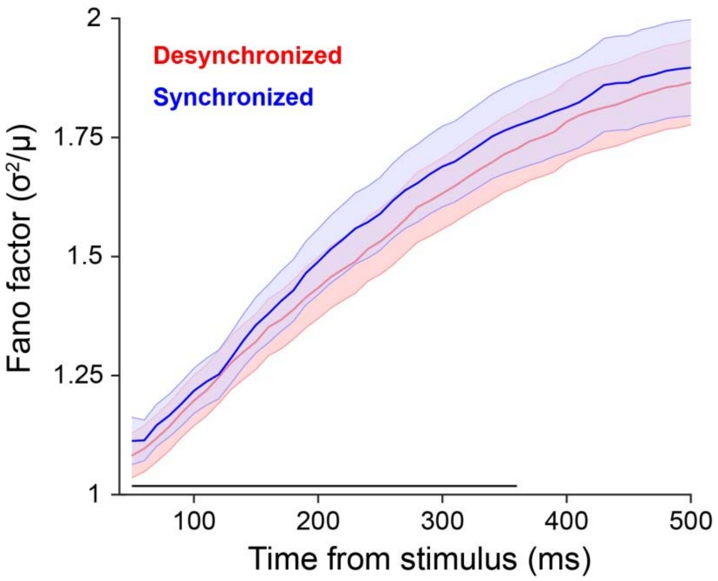

Supplementary Figure 4: Fano factor (FF, variance/mean) in a growing window for synchronized and desynchronized states. FF was plotted as a function of growing window size (50 ms to 500 ms post-stimulus) in each cortical state. There was no significant difference between the two states for any window size ($P > 0.05$).

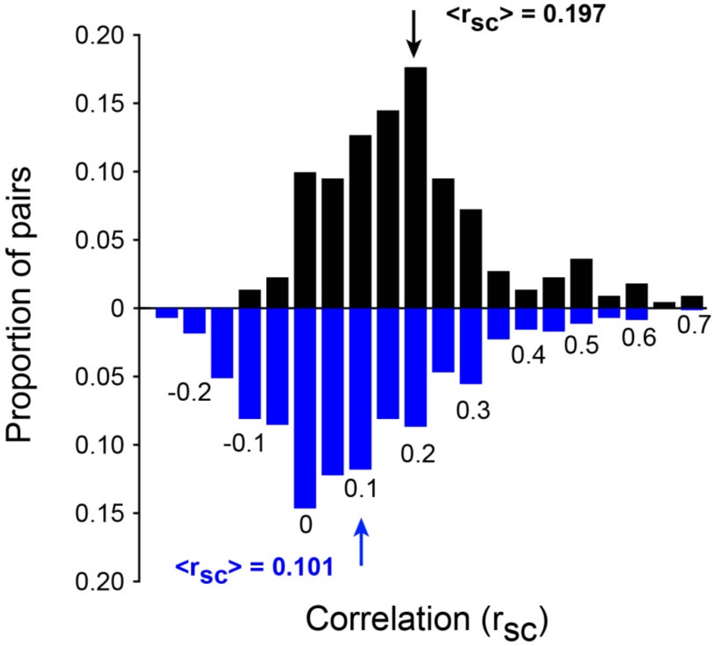

Supplementary Figure 5: Histogram of synchronized (blue) and resting state (black) correlation coefficients. Neuronal responses in the resting state was subdivided into 1 second trial periods in order to calculate the Pearson correlation coefficient for all pairs (n = 221). The arrows represent the mean $<r_{sc}>$ for each group.

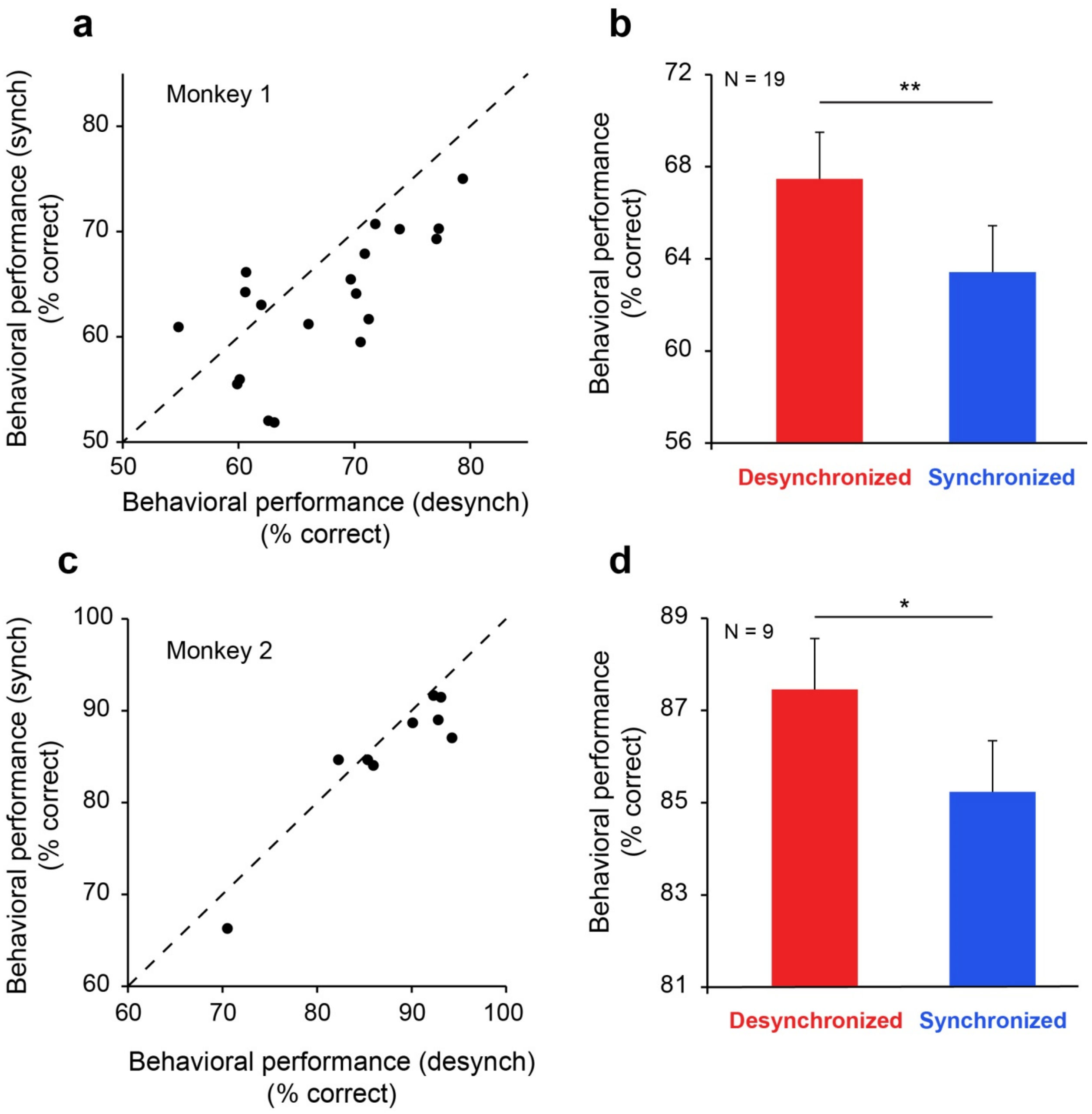

Supplementary Figure 6: Behavioral performance is higher in desynchronized trials for each monkey. (A and C) Scatter plots showing the % correct responses in the synchronized vs. desynchronized trials for monkey 1 (panel A, N = 19 sessions) and monkey 2 (panel C, N = 9 sessions). **(B and D)** Behavioral discrimination performance was significantly greater in the desynchronized trials for monkey 1 (**P < 0.01) and monkey 2 (*P < 0.05).

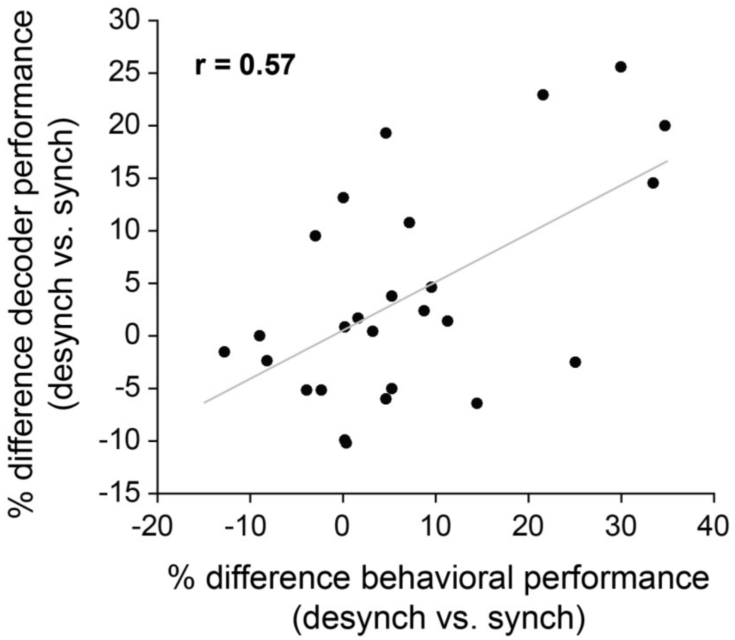

Supplementary Figure 7: Percent difference in linear classifier performance vs. percent difference in behavioral performance (desynch vs. synch). We selected orientations with a large difference between target and test (10-20°) in the synchronized and desynchronized states. There was a significant correlation between the change in classifier performance and change in behavioral performance ($r = 0.5741$, $P = 0.0022$, Pearson correlation).

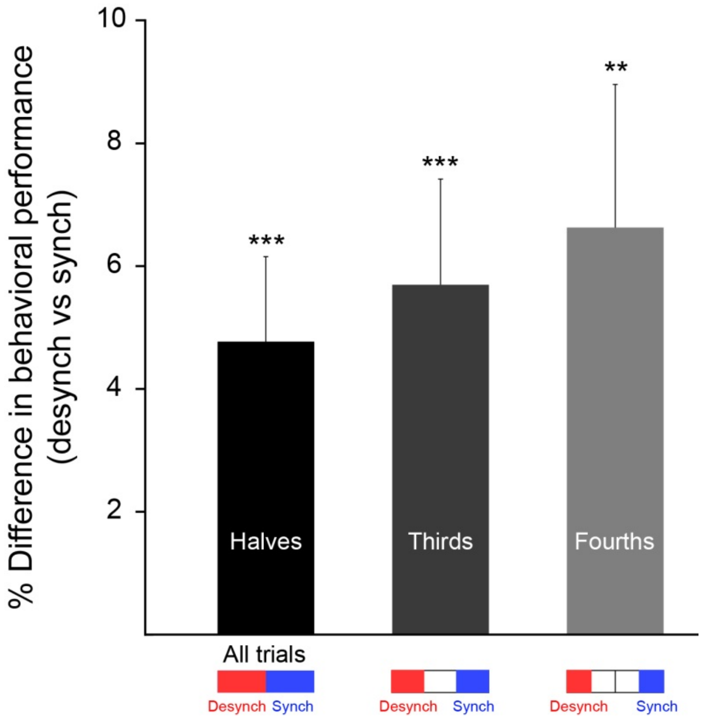

Supplementary Figure 8: Percent performance difference between desynchronized and synchronized trials using different trial separation criteria. "Halves" represents the trial division method implemented throughout the manuscript. That is, we separated all trials based on the median population synchrony index (PSI) value and then compared behavioral performance in the desynchronized vs. synchronized trials (***P < 0.001). 'Red' labels the proportion of desynchronized trials, and 'blue' labels the proportion of synchronized trials chosen for the analysis. "Thirds" represents a classification scheme where we extracted the top 1/3 of the most synchronized trials and the bottom 1/3 of the most desynchronized trials, and then compared neuronal and behavioral effects associated with the two groups of trials (***P < 0.001). "Fourths" represents a classification scheme where we extracted the top 1/4 of the most synchronized trials and the bottom 1/4 of the most desynchronized trials, and then compared the two groups (**P < 0.005).

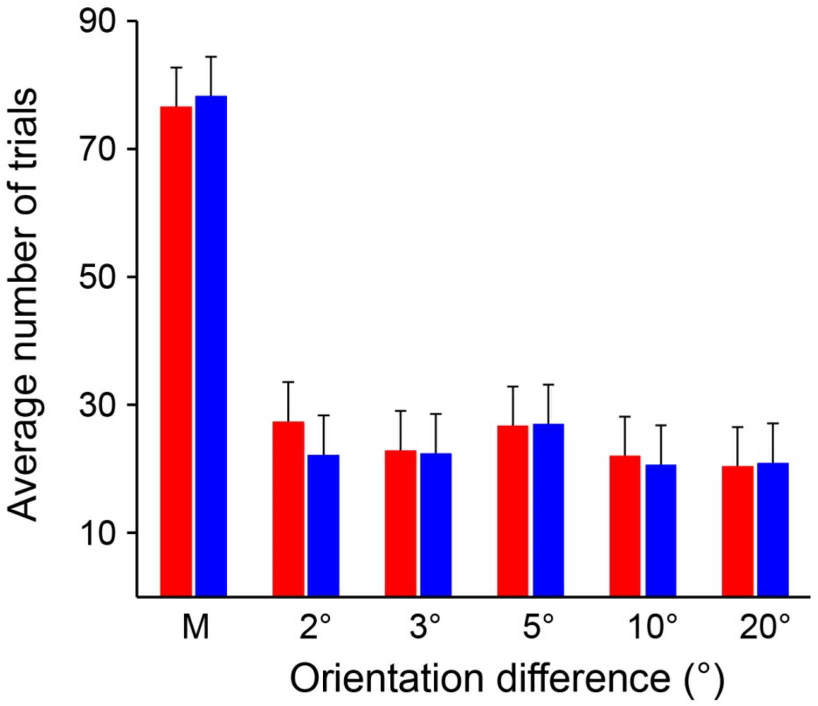

Supplementary Figure 9: Average number of trials (across sessions) corresponding to each test orientation difference for synchronized and desynchronized trials. 'M' stands for 'match' condition (0° orientation difference).

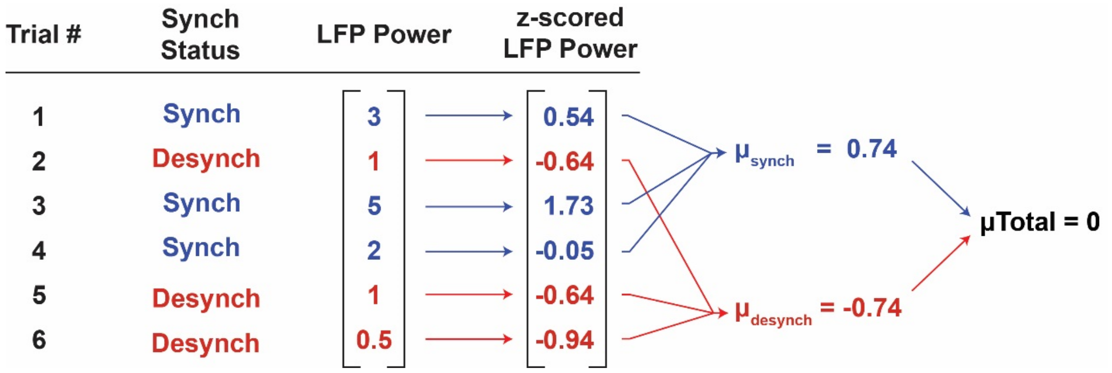

Supplementary Figure 10: Example of 6 trials in which we used z-score analysis for LFP power. We z-scored the LFP power across trials before splitting them into synchronized and desynchronized groups. We then computed the mean z-scored LFP power in each cortical state. In this case, the mean of both groups will always be zero (thus reflecting the imposed symmetry around zero).

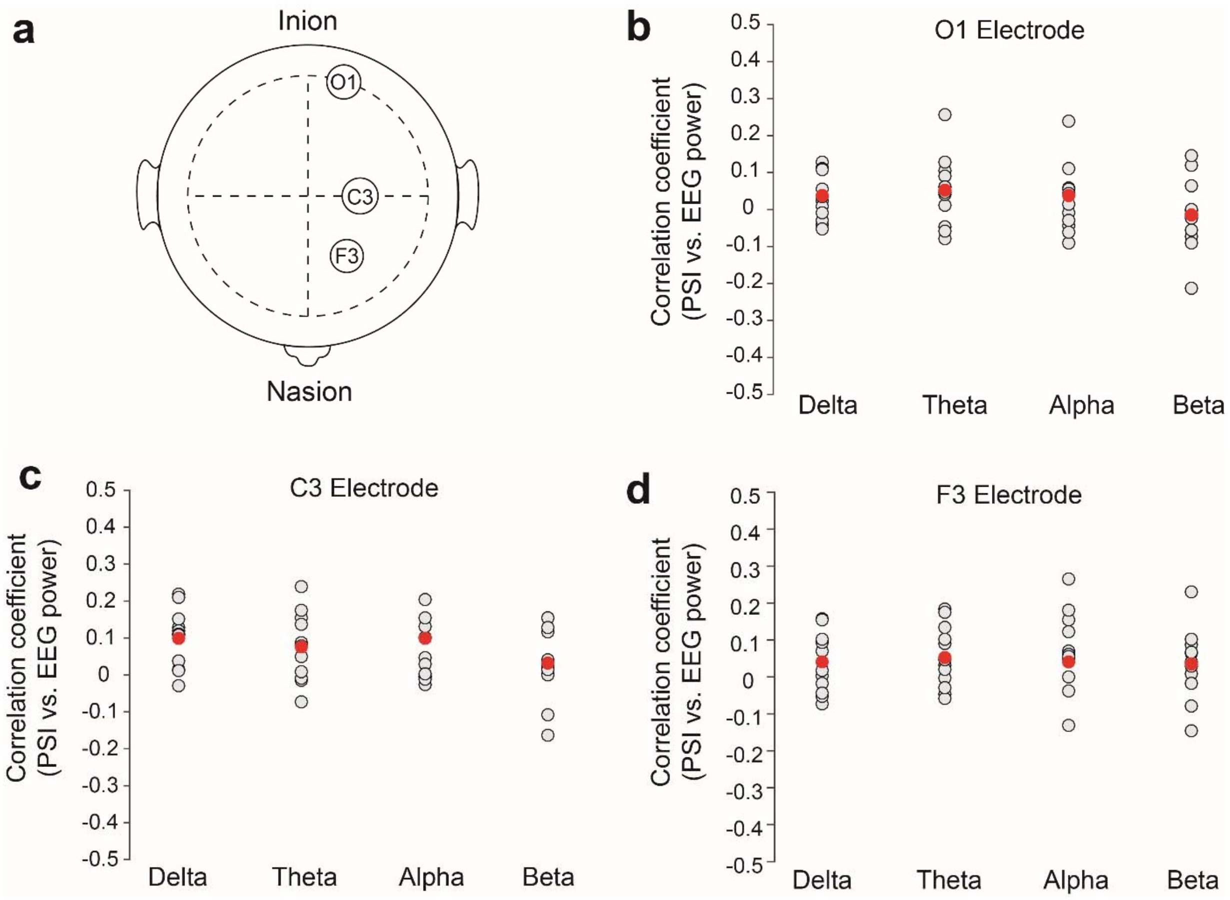

Supplementary Figure 11: Relationship between Population synchrony index and EEG power. (a) 6 mm cast silver, gold-plated, cup electrodes (Grass Technologies) were attached to an elastic cap fitted to each monkey over the international standard 10-20 system of EEG sites corresponding to F3, C3 and O1. (b) Correlation coefficient between PSI and EEG power for each physiological band for the O1 electrode. $\mu r_{delta} = 0.038 \pm 0.019$, $\mu r_{theta} = 0.051 \pm 0.027$, $\mu r_{alpha} = 0.027 \pm 0.025$, $\mu r_{beta} = -0.014 \pm 0.028$. (c) Correlation coefficient between PSI and EEG power for each physiological band for the C3 electrode. $\mu r_{delta} = 0.099 \pm 0.022$, $\mu r_{theta} = 0.076 \pm 0.026$, $\mu r_{alpha} = 0.061 \pm 0.022$, $\mu r_{beta} = 0.033 \pm 0.029$. (d) Correlation coefficient between PSI and EEG power for each physiological band for the F3 electrode. $\mu r_{delta} = 0.042 \pm 0.023$, $\mu r_{theta} = 0.054 \pm 0.024$, $\mu r_{alpha} = 0.070 \pm 0.030$, $\mu r_{beta} = 0.035 \pm 0.027$.

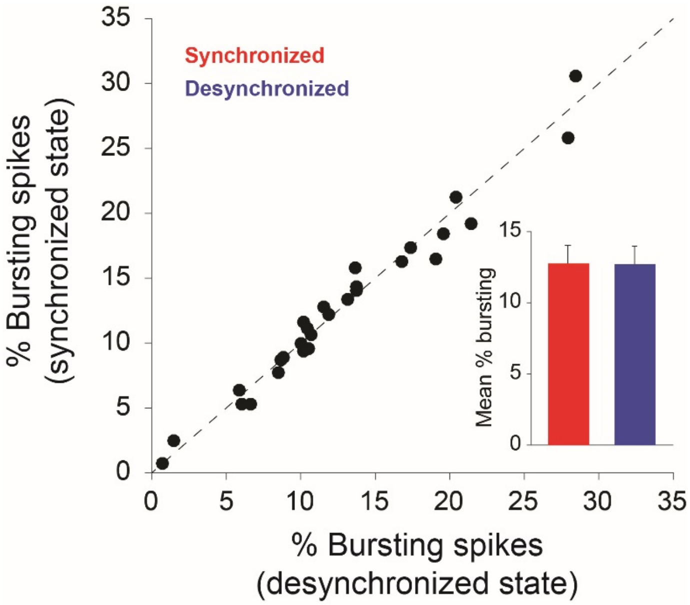

Supplementary Figure 12: Percentage of bursting spikes in synchronized and desynchronized states. We computed the percentage of spikes with an interspike interval less or equal to 5 ms on individual trials in both the synchronized and desynchronized states for each session. We found no significant difference in bursting between the two conditions across sessions (% $\text{Bursting}_{desynch}$ = 12.76 ± 1.27 vs. % Bursting_{synch} = 12.71 ± 1.26) (p = .81, paired t-test).

AUTHOR INFORMATION

Charles B. Beaman[1], Sarah L. Eagleman[1,2], and Valentin Dragoi[1]

[1] Department of Neurobiology and Anatomy
McGovern Medical School, University of Texas at Houston
Houston, TX 77030

[2] Department of Electrical and Computer Engineering
Rice University, George R. Brown School of Engineering
Houston, TX 77005

The authors have no competing financial interests to declare.
